# The anti-oncogenic effect of 17-DMAG via the inactivation of HSP90 and MET pathway in osteosarcoma cells

**DOI:** 10.32604/or.2023.029745

**Published:** 2023-07-21

**Authors:** MASANORI KAWANO, KAZUHIRO TANAKA, ICHIRO ITONAGA, TATSUYA IWASAKI, YUTA KUBOTA, HIROSHI TSUMURA

**Affiliations:** Department of Orthopaedic Surgery, Faculty of Medicine, Oita University, Oita, 879-5593, Japan

**Keywords:** Osteosarcoma, MET, HSP90, 17-DMAG

## Abstract

Heat shock protein (HSP) 90 plays a crucial role in correcting the misfolded three-dimensional structure of proteins, assisting them in folding into proper conformations. HSP90 is critical in maintaining the normal functions of various proteins within cells, as essential factors for cellular homeostasis. Contrastingly, HSP90 simultaneously supports the maturation of cancer-related proteins, including mesenchymal epithelial transition factor (MET) within tumor cells. All osteosarcoma cell lines had elevated MET expression in the cDNA array in our possession. MET, a tyrosine kinase receptor, promotes proliferation and an anti-apoptotic state through the activation of the MET pathway constructed by HSP90. In this study, we treated osteosarcoma cells with an HSP90 inhibitor, 17-demethoxygeldanamycin hydrochloride (17-DMAG), and assessed the changes in the MET signaling pathway and also the antitumor effect of the drug. The cell cycle in osteosarcoma cells administered 17-DMAG was found to be halted at the G2/M phase. Additionally, treatment with 17-DMAG inhibited cell proliferation and induced apoptosis. Inhibition of tumor cell proliferation was also observed in an *in vivo* model system, mice that were treated with 17-DMAG. Based on the results of this study, we were able to confirm that 17-DMAG promotes inhibition of osteosarcoma cell proliferation and induction of apoptosis by inhibition of MET, a protein highly expressed in osteosarcoma cells. This approach may be useful for the establishment of a new treatment strategy for patients resistant to the standard treatment for osteosarcoma.

## Introduction

Osteosarcoma (OS) is the most common type of primary bone cancer, and the standard treatment protocol involves a combination of chemotherapy and surgery [1]. Despite the advances in chemotherapy, patients with osteosarcoma demonstrate poor prognosis due to metastases to the lung and other organs. Additionally, the drugs used in chemotherapy are mainly anti-cancer drugs that have been in use since the 1970s [2]. Although various molecular-targeted drugs have been developed for treatment of other types of cancers, no new drugs have been developed for treatment of malignant bone cancers.

Heat shock protein 90 (HSP90) is a molecular chaperone with tyrosine kinase activity and is involved in the transport and activation of various proteins in the cell [3,4]. In malignant tumors, HSP90 causes failure of various control mechanisms. Therefore, HSP90 inhibitors (HSP90i), multi-kinase inhibitors, are gaining attention as potential anti-cancer drugs [5,6]. HSP90i are also superior in terms of selectivity, as HSP90 demonstrates higher activity levels in tumor cells than in normal cells.

We assessed four osteosarcoma cell lines and a control fibroblast cell line using microarray profiling and identified the factors that were elevated in the osteosarcoma cell lines. MET, an HSP90 client protein, was found to be elevated in all the osteosarcoma cell lines. MET is an oncogenic gene that encodes c-MET, a receptor tyrosine kinase, that uses hepatocyte growth factor as a ligand [7]. The hepatocyte growth factor/c-MET signaling pathway aids tumor formation by promoting cell proliferation, survival, and motility [8,9]. It has previously been reported that HSP90, a protein chaperone, forms a complex with MET in cancer cells, and treatment of the cells with 17-demethoxygeldanamycin hydrochloride (17-DMAG) results in the ubiquitination of MET [10,11]. The MET protein is stabilized and exerts its kinase activity when in complex with HSP90 and p23 proteins [12,13]. However, when p23 is removed and CHIP protein is added to the complex, the MET protein is modified with ubiquitin and destroyed by the proteasome [14,15]. The binding of p23 and CHIP to the MET-HSP90 complex was examined by administration of 17-DMAG.

The MET pathway is also involved in tumor growth and metastasis progression in osteosarcoma [16,17]. Based on the microarray results, we found that MET potentially plays an important role in osteosarcoma. If the activation of the MET pathway can be inhibited by 17-DMAG, an HSP90 inhibitor, the proliferative capability and anti-apoptotic state of osteosarcoma cells may be altered, resulting in an antitumor effect. In this study, we aimed to assess the antitumor effect of 17-DMAG in osteosarcoma cells through the inhibition of MET tyrosine kinase activity and the downstream signaling function. Thus, the results of this study may aid in the development of new treatment strategies for patients with osteosarcoma resistant to the standard treatment procedures.

## Materials and Methods

### Cell culture

The human OS cell lines—HOS, SaOS, and MG-63—were obtained from RIKEN Cell Bank (Tsukuba, Japan), and NY and MRC5 were obtained from JCRB Cell Bank (Osaka, Japan). The genotype and phenotype of each cell line was authenticated by the respective source company. HOS cells were grown in minimal essential medium (MEM) supplemented with 10% fetal bovine serum (FBS; Invitrogen, NY, USA) and 0.1 mmol/L nonessential amino acids (NEAA). SaoS, MG-63, NY and MRC5 cells were cultured in a high-glucose medium, Dulbecco’s modified eagle medium (DMEM) (Invitrogen, NY) supplemented with 10% FBS and 1% penicillin and streptomycin. The cells were incubated at 37°C in an incubator chamber supplemented with 5% CO_2_ and passaged when the cells were grown to approximately 70% confluent, as previously described [18].

### Analysis of mRNA expression using cDNA arrays

We analyzed the mRNA of four osteosarcoma cell lines using a cDNA microarray, using MRC5 as the control cell line. The GeneChip Genome HG U133 Plus 2.0 Array (Affymetrix, Tokyo, Japan) was used for mRNA expression profiling of the cell lines, as previously described [19]. The gene list was filtered with a fold-change cutoff of 2, i.e., we obtained a list of genes with significant differential expression of two folds or more. The cDNA array data was registered in Gene expression Omnibus (GEO). You can access our data for mRNA. Our GEO reference number is GSE70415 and URL to access our raw data is below: http://www.ncbi.nlm.nih.gov/geo/query/acc.cgi?acc=GSE70415.

### Immunoprecipitation

Cells were cultured on 6-well plates and harvested and solubilized in lysis buffer (Pierce IP lysis buffer; Thermo Scientific, Rockford, IL, USA). After centrifugation for 1 min at 8000 rpm at RT in a microcentrifuge, 200 ml supernatant (1 mg/ml) was incubated for 12 h at 4°C with 2 μg anti-HSP90 (ab59459; Abcam, Tokyo, Japan) and anti-MET (#3148; Cell Signaling Technology (CST), Tokyo, Japan) antibodies. Following the addition of 30 µL of Protein G Sepharose® 4 beads (GE Healthcare, Tokyo, Japan), the mixture was incubated for 2 h, at 4°C with rotation. The immune complex was washed three times with lysis buffer, and the sepharose beads were then boiled for 10 min in the sample buffer. The immunoprecipitate was run on Sodium dodecyl sulfate (SDS)-Polyacrylamide gel electrophoresis (PAGE) by using 4%–20% gradient precast gels (Bio-Rad). Western blotting was then performed with primary antibodies against CHIP (#2080; Cell Signaling Technology (CST), Tokyo, Japan), p23 (ab226295; Abcam, Tokyo, Japan), HSP90 (#4877; CST), MET (ab51067), GAPDH (#5174; CST), and ubiquitin (#3936; CST). 50 mM MG132 was added to osteosarcoma cells for 90 min as a positive control for ubiquitination. Phosphate-buffered saline (PBS) was added to the cells as negative control.

### Knockdown of HSP90 and MET expression using siRNA

small interfering RNA (siRNA) oligonucleotides targeting HSP90 (Assay ID: 121532) and MET (Assay ID: 103551) mRNA was purchased from Ambion (Tokyo, Japan) and MISSION siRNA Universal Negative Control (*SIC* 001) was purchased from Sigma-Aldrich (Osaka, Japan), as previously described [20] The siRNAs at 40 nM were transfected into osteosarcoma cells using Lipofectamine 2000 reagent according to the manufacturer’s instructions. The cell lines were harvested 48 h at 37°C in a CO_2_ incubator after the transfection, then subjected to various analyses.

### MTT assay and cell proliferation analysis

The osteosarcoma cell lines and MRC5 were cultured in 6-well plates in 2 mL medium in the absence of antibiotics. The cells were treated with 17-DMAG at varying concentrations (0, 5, 10, 50,100, 500, and 1000 nM) for 24 h. The treated cells were then cultured in 96-well plates at a seeding density of 2 × 10^3^ cells per well for 24 h. The cells were stained with 10 µL of 5 mg/mL 3-[4,5-dimethylthiazol-2-yl]-2,5-diphenyltetrazolium bromide (MTT) dye per well (Sigma Aldrich; Darmstadt, Germany) for 4 h at 37°C. The culture medium was then discarded, and 150 µL of dimethyl sulfoxide was added. The absorbance of blue formazan crystals was measured at 570 nm using an Enzyme Linked Immuno-Sorbent Assay (ELISA) plate reader (Multiskan FC; Thermo Scientific, Germany). The osteosarcoma cell lines and MRC5 (1 × 10^5^ cells per well) were cultured in 6-well plates in 2 mL medium without antibiotics. The cells were treated with 17-DMAG at different concentrations (0, 10, 25, 50, 100, 200, 400, and 1000 nM) for 12, 24, and 48 h, respectively. The number of viable cells present at the end of the experiment was counted using a TC10 Automated Cell Counter (Bio-Rad, Tokyo, Japan). All experiments were performed in triplicate.

### Western blotting

Western blot analysis was adopted from a previous study [18]. Total protein was extracted out of tumor cells with the Radio Immunoprecipitation Assay (RIPA) lysate, and the protein content was determined by the Bicinchoninic Acid (BCA) method. Next, the protein (20 µg/lane) was separated by 10% polyacrylamide gel electrophoresis and transferred to polyvinylidene difluoride membranes. The membranes were then blocked with Blocking One (INC.05999-84, NACALAI TESQUE, Kyoto, Japan) and maintained with the primary antibodies at 4°C overnight. After the membranes were washed with 0.1% Phosphate buffered saline with Tween-20 (PBST), they were incubated with the secondary peroxidase conjugated anti-rabbit secondary antibodies (Jackson ImmunoResearch Laboratories Inc., West Grove, PA, USA) at room temperature for 1 h. The strips were developed using an efficient chemiluminescence kit after re-washing with PBST. The primary antibodies included rabbit anti-MET (#8023), phospho (p)-MET (Tyrosine (Y) 1234) (#3077), PI3K (#4252), Akt (#4691), p-Akt (Serine (S) 473) (#4060), β-actin (#4970), CHK1 (#2360S), CCNB1 (#12231S), CDK1 (#28439S), PARP (#9542), cleaved PARP (#9541), Caspase 3 (#9662), cleaved Caspase 3 (#9661), Caspase 7 (#9492), cleaved Caspase 7 (#9491), Caspase 8 (#4790), Cleaved caspase 8 (#9496), RhoA (#2117), ROCK (#4035), Src (#2109), p-Src (S17)(#12432), FAK (#3285), p-FAK (Y397)(#8556), Paxillin (#12065), p-Paxillin (Y118) (#69363), and GAPDH (#5174); all purchased from CST. All the primary antibodies were used at a 1:1000 dilution. Peroxidase-conjugated anti-rabbit IgG secondary antibodies (Jackson ImmunoResearch Laboratories Inc., West Grove, PA, USA) were used at a 1:2000 dilution.

### Cell cycle analysis

The MG63 cells were seeded and incubated for 12 h. The cells were then treated with 17-DMAG (50, 75, 100 nM) and incubated for 12 h. For cell cycle analysis, the MG63 cells were stained with PI using a Cycletest Plus DNA Reagent Kit (BD Biosciences, Tokyo, Japan) following the manufacturer’s protocol. We performed flow cytometric analysis on a BD Fortessa with fluorescence-activated cell sorting (FACS) Diva Software (BD Biosciences, Tokyo, Japan) and analyzed using FlowJo 10.2. The percentages of cells in the G0/G1, S, and G2/M phases were assessed, as previously described [19].

### Detection of apoptosis

Apoptotic cell death was determined by FACS using an Annexin V-FITC apoptosis detection kit (BD Biosciences) as previously described [20]. Total apoptosis is indicated as the sum of percentages of early and late apoptosis.

### Cell motility and migration assays

Cell migration and invasion were investigated by Transwell chamber of a 24-well insert (8 µm pore size; BD Biosciences, San Diego, CA, USA) without or with Matrigel. Osteosarcoma cells (5 × 10^4^) were placed in the upper layer of chamber, whereas 20% FBS was added to underlayer of chamber, as previously described [20]. Briefly, the lower compartment was filled with 600 ml of a medium containing 30% FBS as a chemoattractant. After being cultured in 5% CO_2_ at 37°C for 16 h, the inner cells were removed with cotton swabs; then, the outer cells penetrating the chamber were immersed in 75% ethanol for 15 min, followed by 0.4% crystal violet solution for 15 min at room temperature, and finally in distilled water 2–3 min for three times. Then we examined by using g light microscopy.

### Immunofluorescence analysis

Immunohistochemistry was used to evaluate the presence of actin filaments in the cells. After washing with PBS, the rehydrated culture dishes were stained with Alexa 488 conjugated anti-phalloidin antibody (A12379; Invitrogen, Tokyo, Japan), diluted to 1:100 in an Ab Diluent (Dako ChemMate; Dako, Tokyo, Japan), and incubated overnight at room temperature. The mounting medium with 4′6-diamino-2-fenilindol dihidrocloreto (Vector Laboratories, Inc., Newark, USA) was applied to the cells and left to fix for 6 h in a dark room. Digital images were acquired as Z-stack confocal images using a Zeiss 710 confocal microscope and Zen software (Carl Zeiss, Jena, Germany).

### In vivo experiments using nude mice

BALB/c nu/nu mice (6–8 weeks old, female, body weight: 20 ± 5 g), were acquired from the Kyodo Laboratory (Tosu, Japan). After quarantine, all mice were kept in a pathogen free environment on a standard 12 h-day/12 h-night cycle and were fed a standard sterilized pellet diet and water *ad libitum*. They were continuously monitored during daytime from Monday to Friday, and twice daily during daytime on Saturdays, Sundays, and holidays for signs of poor health. Injection of 2 × 10^6^ MG63 cells suspended in 100 μl of normal saline into the gluteal region of BALB/c nu/nu mice was performed Tumors were measured using calipers two or three times per week, as previously described [18]. Our experimental procedures involving mice were performed in accordance with the 3Rs (replace, reduce, and refine) principles and a legal requirement in the European Union Directive 2010/63/EU [21]. The groups included saline treated group and mice were intraperitoneally injected with 17-DMAG (D5193; Sigma Aldrich, Tokyo, Japan) at a dose of 25 mg/kg three times a week. The changes in tumor volume were monitored for four weeks. The volume of the primary tumor was calculated using the formula: Volume = (Length × Width^2^)/2. Lung metastatic tumors were measured using a micro-CT apparatus (R_mCT) which allows us to obtain high-resolution CT images in small living animals. The tumor volume of the lung nodule was estimated using the formula: (π × long axis × short axis × short axis)/6. After 4 weeks, the mice were then sacrificed by cervical dislocation and xenografted tumors were resected and subjected to immunohistochemistry. After conventional paraffin embedding and sectioning (4 μm), xenografted tumor tissues were routinely dewaxed in xylene, hydrated in gradient alcohol, and inactivated by 3% hydrogen peroxide for 10 min. Microwave repair (Hydrogen ion concentration = 6.0, 15 min) was performed by applying 0.01 mol/L sodium citrate buffer. After the sections were closed with 5% bovine serum albumin (Wako, Tokyo, Japan) for 20 min at room temperature, the primary antibody anti-RhoA (ab54835; Abcam) and p-Src (Y527) (#2105; CST) were added dropwise and maintained overnight at 4°C. The next day, the goat anti-rabbit secondary antibody was added dropwise and maintained for 20 min at room temperature. The detection of the proteins was performed by EnVisionTM Detection System, Peroxidase/DAB, Rabbit/Mouse (Dako, Tokyo, Japan). Tris buffer (pH 7.6) was used for washing between the various steps. Nuclei of all samples were counterstained with haematoxylin. The samples were then dehydrated and cover-slipped. For image analysis, the RGB images of different 5 fields of vision obtained by light microscope at magnification 400× for each sample.

### Statistical analysis

A two-tailed Student’ s *t*-test was carried out for continuous variables. The differences among more than three groups were analyzed using analysis of variance (ANOVA) and the Scheffe’s test. The results were expressed as the mean ± standard deviation (SD), and the differences were considered significant when the *p* value was less than 0.05. All statistical analyses were performed using SPSS 24.0 software (IBM, Tokyo, Japan).

## Results

### Analysis of mRNA expression by cDNA arrays

**Figure 1:**
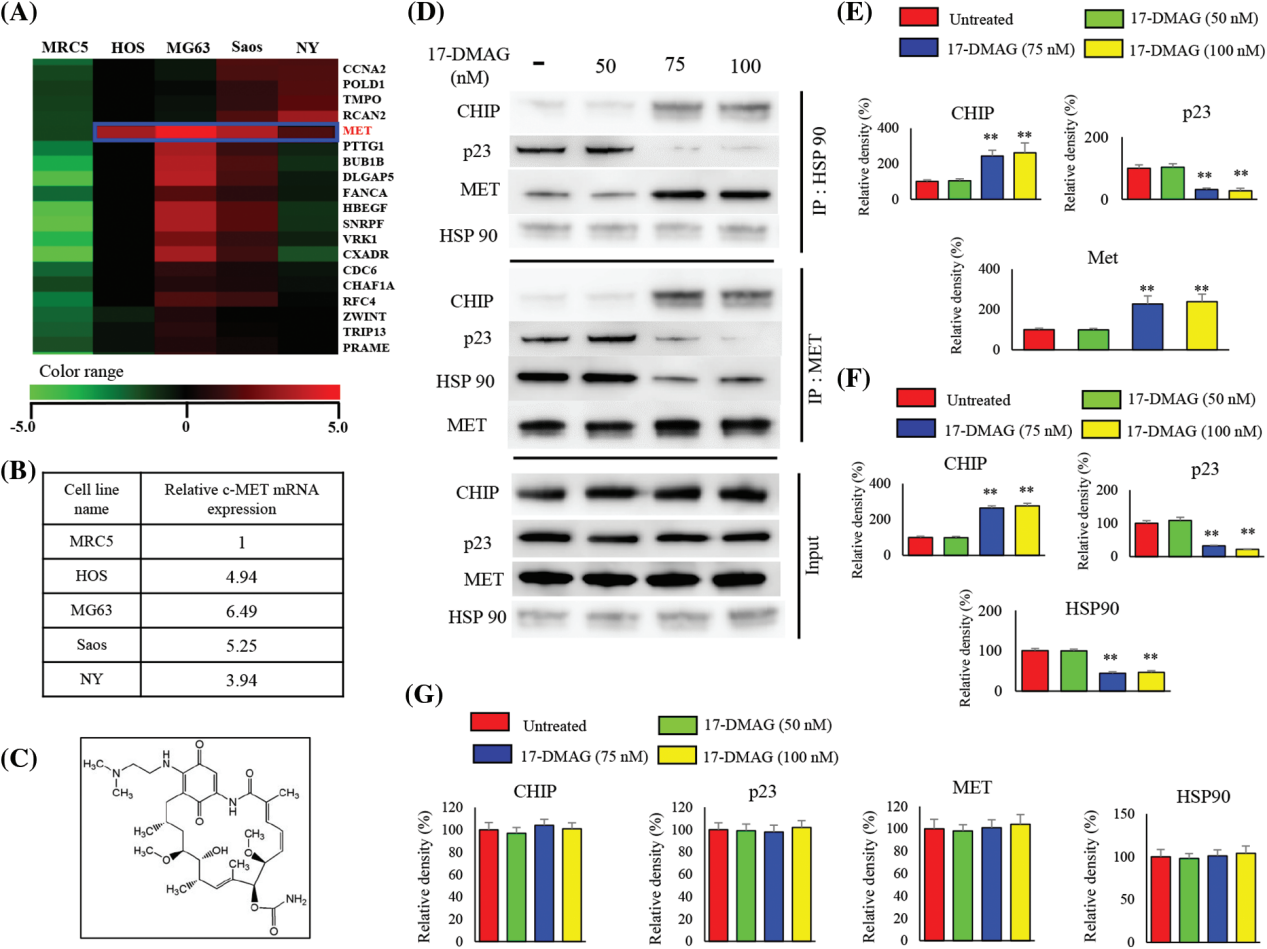
cDNA microarray and effects of 17-DMAG on the HSP90-MET complex. Expression of mRNA in four osteosarcoma cell lines compared with that in the control MRC5 cell line. MET expression was uniformly upregulated in the osteosarcoma cell lines when compared with the expression in MRC5 cells (A). Relative expression of MET in osteosarcoma cells compared with that in MRC5 (B). Chemical structure of 17-DMAG (C). The HSP90-MET complex is transformed by 17-DMAG in such a way that it binds to CHIP instead of p23 (D). Administration of 17-DMAG resulted in an increase in the expression of CHIP, while p23 expression decreased (E). Immunoprecipitation was performed using the MET antibody, and changes in protein expression resulting from the formation of the MET-HSP90 complex were investigated (F). Administration of 17-DMAG resulted in an increase in the expression of CHIP, while p23 expression decreased (G). **p < 0.01.

There were 745 genes with an increase of two-fold or more and 241 genes with a decrease of 50% or more, common to all the osteosarcoma cell lines compared with the control MRC5 cell line (Fig. 1A). Increased expression of MET was also observed across all four osteosarcoma cell lines compared with MRC5 cell line. The expression of MET mRNA in HOS, MG63, Saos, and NY cells was up-regulated to 4.94-, 6.49-, 5.25-, and 3.94-fold of that in the MRC5 cells, respectively (Fig. 1B). By positioning MRC5 as normal cells, we searched for the factors that show elevated mRNA expression common to the four types of osteosarcoma cells. We identified c-MET as a factor that showed an increase of more than twice that of MRC5.

### Effect of 17-DMAG on MET-HSP90 complex

Immunoprecipitation using an HSP90 antibody was performed with the extract from osteosarcoma cells after treatment using 17-DMAG (Fig. 1C), and immunoblotting was then performed with the pulled-down proteins (Fig. 1D). Cells treated with 17-DMAG at doses of 75 nM and higher exhibited an increase in the expression of CHIP, a ubiquitin ligase, and a decline in the expression of p23, an HSP90 co-chaperone protein (Fig. 1E). Immunoprecipitation was then performed using the MET antibody, and immunoblotting was again performed with the pulled-down proteins (Fig. 1F). In the groups administered 17-DMAG, an increase in CHIP expression, and a decline in p23 and HSP90 protein expression were similarly observed (Fig. 1G).

### 17-DMAG affects ubiquitination and phosphorylation of MET protein

**Figure 2:**
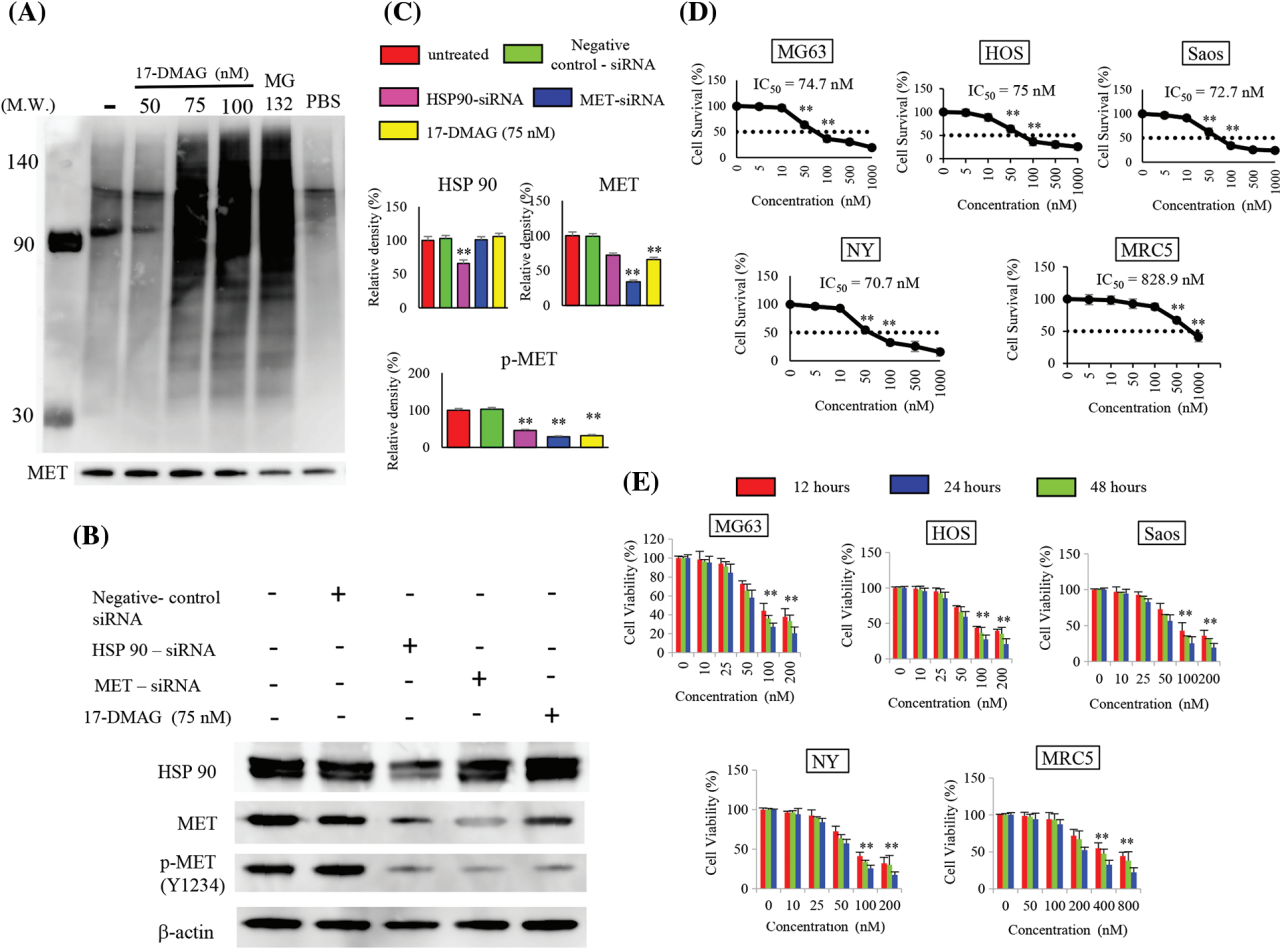
Effect of 17-DMAG on target protein expression and the growth of osteosarcoma cells. MET exhibits correct folding by HSP90, but inhibition of HSP90 leads to MET ubiquitination. Immunoprecipitation was performed using the MET antibody, and ubiquitin expression was assessed. Administration of 17-DMAG resulted in MET ubiquitination. MG-132 at a 50 µM concentration served as a positive control. PBS administration was as negative control. (A) Comparison of HSP90 and MET expression inhibition by siRNA, and HSP90 expression inhibition using 17-DMAG: Knockdown of HSP90 expression inhibited the expression of HSP90, MET, and phosphorylated (p)-MET. Knockdown of MET expression inhibited MET and p-MET (Y1234) expression but had no effect on HSP90 expression. (B) Treatment of cells with 17-DMAG did not have any major effects on HSP90 or MET expression, but significantly reduced the expression of p-MET (Y1234). (C) Administration of 17-DMAG induced an anti-proliferative effect in osteosarcoma cells and MRC5 cells in a dose-dependent manner. (D) Administration of 17-DMAG induced an anti-proliferative effect in osteosarcoma cells and MRC5 cells in a time-dependent manner (12, 24, and 48 h). (E) All data represent the mean ± SD from three independent experiments. **p < 0.01.

When immunoblotting with the ubiquitin antibody after IP with MET antibody was performed with this extract, polyubiquitination was observed when cells were treated with 17-DMAG at doses of 75 nM or higher (Fig. 2A). Inhibition of HSP90 by 17-DMAG and knockdown of HSP90 using siRNA both suppressed the phosphorylation of MET protein in the same way. HSP90-siRNA resulted in a minor decrease in MET protein expression but was coupled with a significant decline in MET phosphorylation. MET-siRNA had no effect on the expression of HSP90, but a decline in the expression of MET and MET phosphorylation was observed. Treatment of cells with 17-DMAG had no effect on the expression of HSP90 protein, but the total and phosphorylation of MET decreased significantly (Fig. 2C).

### 17-DMAG inhibits proliferation of osteosarcoma cell lines

We treated the osteosarcoma cell lines (HOS, Saos, MG63, Saos, NY) with 17-DMAG, and investigated the effect on cell viability and proliferation. The concentrations of 17-DMAG required for 50% growth reduction in the survival curve (i.e., IC50 value) of MG63, Saos, HOS, NY and MRC5 were determined to be 74.7, 72.7, 75, 70.7 and 828.9 nM, respectively (Fig. 2D). We also investigated the changes in proliferation in each of the cell lines in a dose- and time-dependent manner. After 48 h of treatment with 100 nM 17-DMAG, the growth ratio of MG63 (27.3% ± 4.1%), Saos (25.6% ± 8.8%), HOS (27.5% ± 6%), and NY (25.6% ± 4.2%) cell lines significantly decreased compared with the growth ratio of MG63 (44.3% ± 7.9%), Saos (42.9% ± 11.2%), HOS (43.7% ± 1.9%), NY (41.1% ± 4.9%) and MRC5 (94.4% ± 8.8%) assessed after 12 h of treatment. Thus, treatment with 17-DMAG significantly inhibited the proliferation of the assessed osteosarcoma cell lines (Fig. 2E).

### Administration of 17-DMAG inhibits activation of MET protein

**Figure 3:**
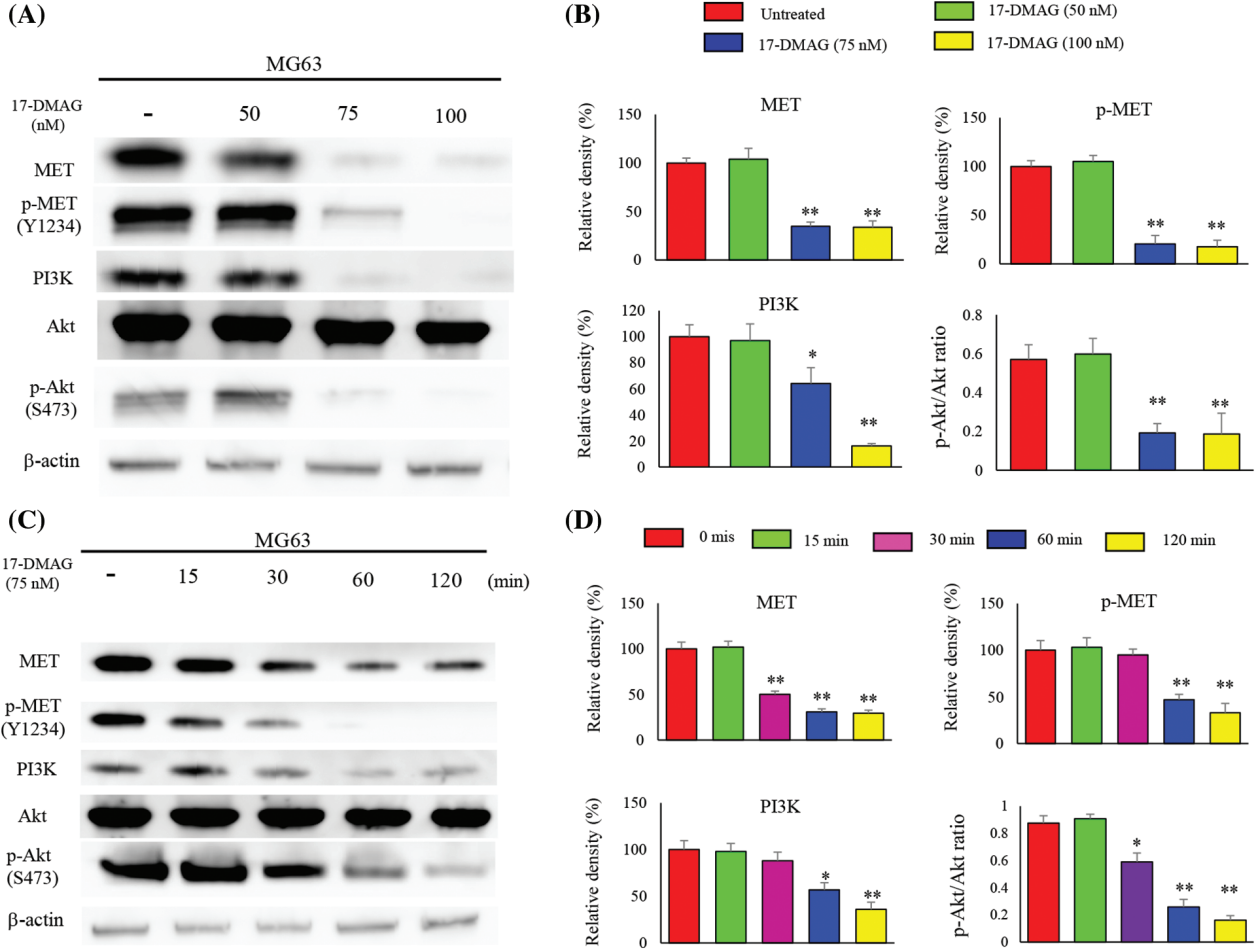
Influence of 17-DMAG on MET protein expression and the downstream signaling pathway. (A) Dose-dependent effects of 17-DMAG on MET and the downstream target protein expression in MG63 cells. (B) Quantification of western blotting. pAKT/AKT ratio was analyzed as quotient of pAKT (S473) *vs*. AKT expression. The data represent the mean ± standard deviation (SD) of three independent experiments. (C) Time-course effects of 17-DMAG on MET and the downstream target protein expression in MG63 cells. (D) Quantification of western blotting. pAKT/AKT ratio was analyzed as quotient of pAKT (S473) *vs*. AKT expression. All data represent the mean ± SD from three independent experiments. *p < 0.05; **p < 0.01.

MET, an HSP90 client protein, was found to be uniformly upregulated in the four osteosarcoma cell lines. Hence, we examined the effect of 17-DMAG in a dose-and time-dependent manner on the expression of MET by western blotting (Fig. 3A). MG63 cells administered 75 nM 17-DMAG showed a decline in the expression of phosphorylated (p)-MET (Y1234) (20.5% ± 8.6%), compared with those administered 50 nM 17-DMAG (105% ± 6.3%). Administration of 17-DMAG significantly reduced Akt phosphorylation and PI3K expression at 75 nM (Fig. 3B). We then determined whether the reduction in the phosphorylation of MET was reaction time-dependent (Fig. 3C). MG63 cells administered 17-DMAG for 60 min showed a decrease in the expression of p-MET (Y1234) (47.3% ± 5.7%), compared those administered the drug for 30 min (95.4% ± 6.2%). Thus, administration of 75 nM 17-DMAG to MG63 osteosarcoma cells inhibited the activation of MET protein, Akt phosphorylation and PI3K expression (Fig. 3D).

### 17-DMAG suppresses cell cycle progression

**Figure 4:**
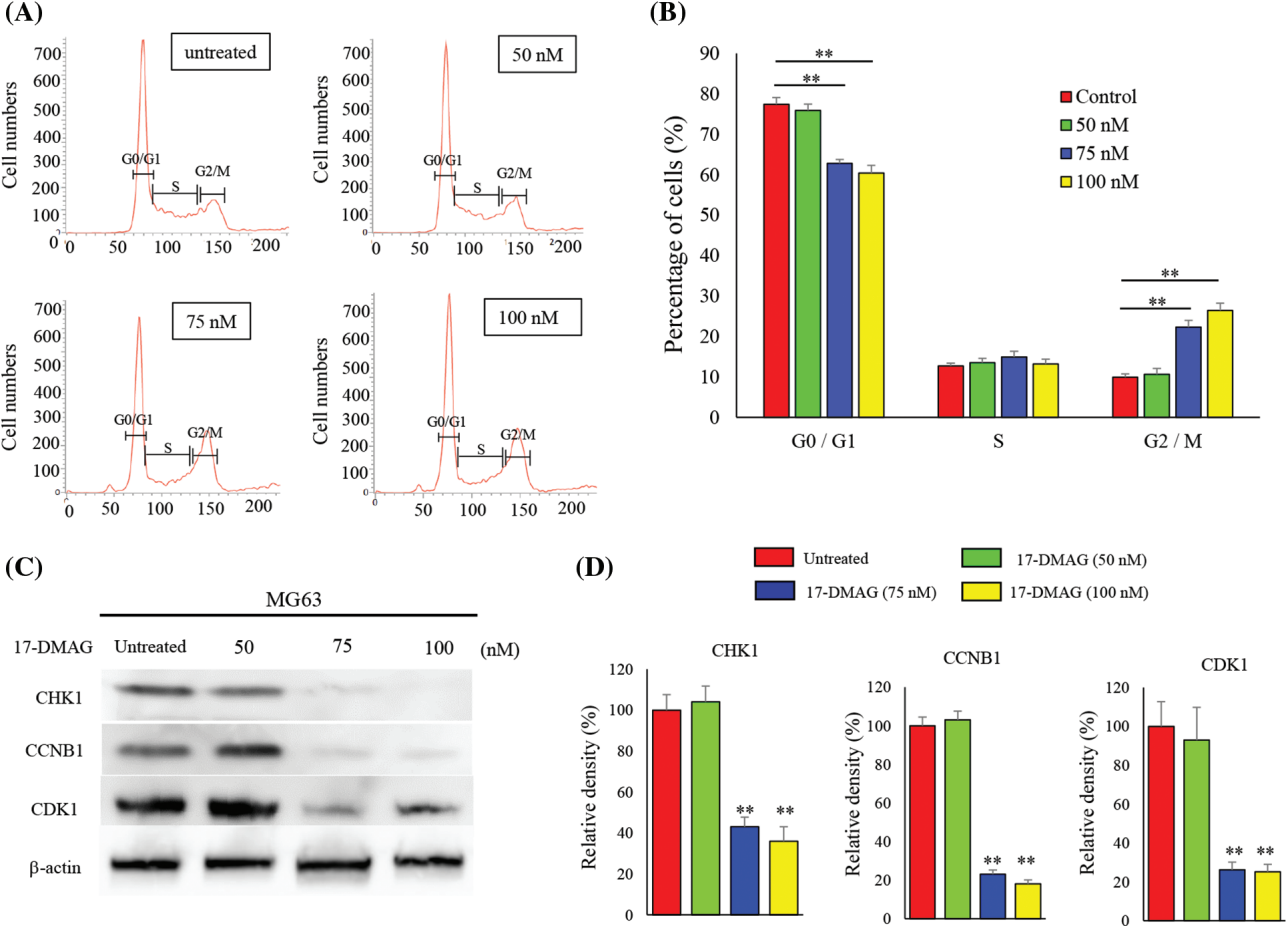
Treatment of osteosarcoma cells with 17-DMAG induces cell cycle retardation. (A) Cells were treated with 17-DMAG at 50, 75, and 100 nM. An untreated control was also included in the experiment. MG63 cells were stained with propidium iodide and analyzed for cell cycle distribution. (B) Histograms showing the mean values of each cell cycle phase. (C) Expression of cell cycle factors associated with the G2/M phase. (D) Quantification of western blotting. All data represent the mean ± SD from three independent experiments. *p < 0.05; **p < 0.01.

We determined whether the observed inhibition of the growth of osteosarcoma cells was mediated by the retardation of cell cycle or induction of apoptosis. Hence, the cell cycle distribution of 17-DMAG-treated cells was analyzed after 12 h (Fig. 4A). We observed that the proportion of cells in the G2/M phase increased, and progression to the S phase was halted for the cell cycle in the 75 nM treatment group. The proportion of cells in the G0/G1 phase was significantly reduced in the treatment groups compared with the untreated group. The proportion of cells in the G2/M phase was significantly increased in the treatment groups compared with group. Based on these results, we concluded that 17-DMAG potentially retarded the cell cycle at the G2/M phase when administered at a dose of 75 nM or higher (Fig. 4B). Next, we investigated the expression of proteins involved in the progression of the cell cycle to the G2/M phase (Fig. 4C). The expression of CHK1, CCNB1, CDK1 were significantly reduced in the treatment groups when compared with the untreated group. Hence, treatment of osteosarcoma cells with 17-DMAG suppressed cell cycle progression (Fig. 4D).

### 17-DMAG accelerates apoptosis-associated protein expression and induces apoptosis in osteosarcoma cells

**Figure 5:**
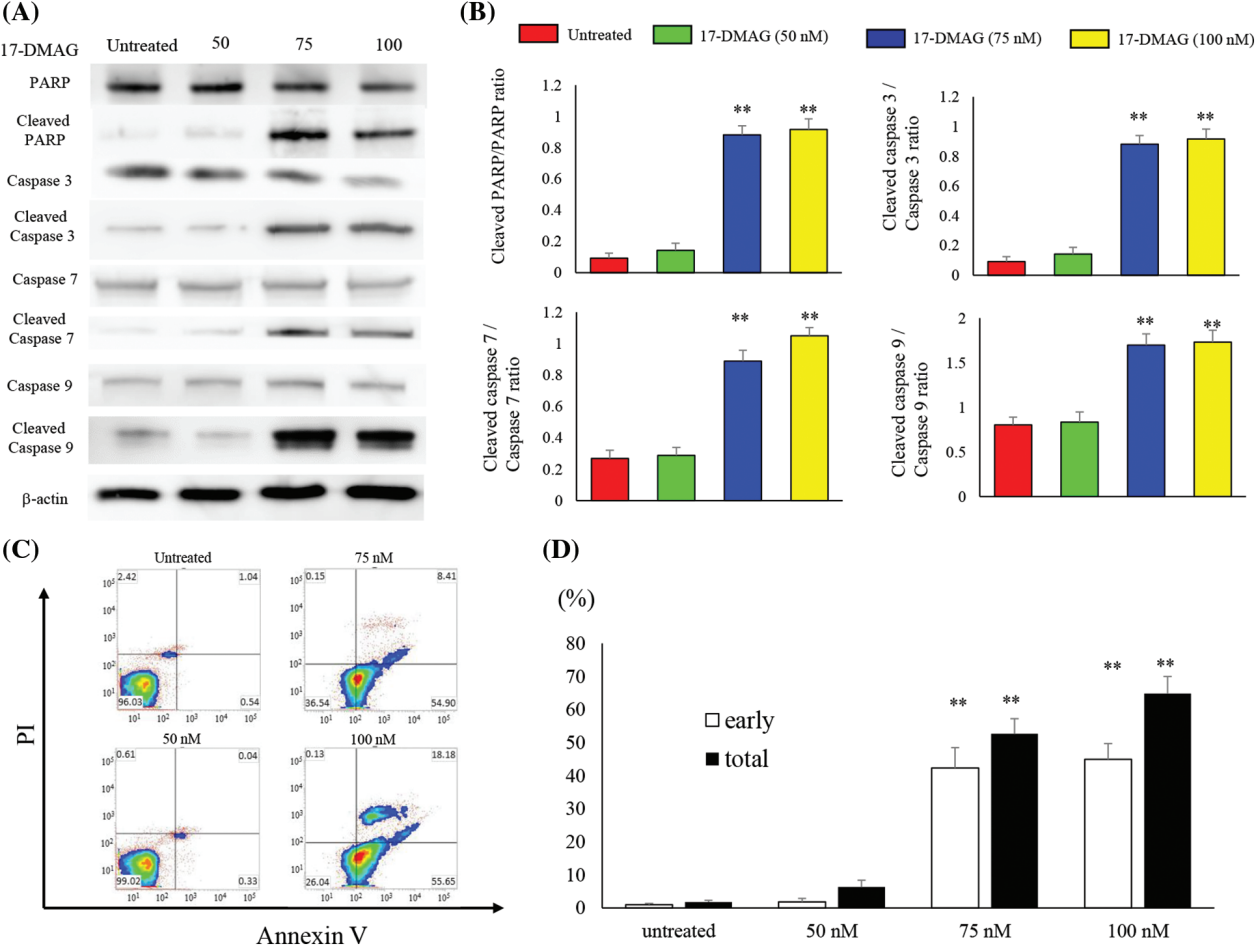
Treatment of osteosarcoma cells with 17-DMAG induces cellular apoptosis. (A) Effect of treatment with 17-DMAG on apoptosis-associated factors analyzed by western blotting. (B) Quantification of western blotting. Cleaved/total ratio was analyzed as quotient of cleaved caspase *vs*. total caspase expression. (C) MG63 cells treated with 17-DMAG were stained with Annexin V-FITC/PI and analyzed for cell apoptosis. (D) Representative percentages of apoptotic cells, where the population of cells stained by Annexin V-FITC(+)/PI(−) and Annexin V-FITC(+)/PI(+) were indicated as early and late apoptosis, respectively. All data represent the mean ± SD from three independent experiments. *p < 0.05; **p < 0.01.

We analyzed the expression of proteins associated with apoptosis in osteosarcoma cells treated with varying doses of 17-DMAG for 24 h (Fig. 5A). No significant difference in poly (ADP-ribose) polymerase (PARP), caspase 3, 7 and 9 expressions were observed among the untreated (100%), 50 nM (99.5% ± 9.2%), 75 nM (85.2% ± 12.8%), and 100 nM (79.9% ± 12%) treatment groups. However, the expressions of cleaved PARP, cleaved caspase 3, 7 and 9 were significantly increased in the treatment groups compared with the untreated group (*p* < 0.01) (Fig. 5B). Furthermore, in flow cytometry analysis using Annexin V-FITC/propidium iodide (PI) double staining, induction of apoptosis was observed in the 75 and 100 nM treatment groups compared to the untreated and 50 nM treatment groups. Administration of 17-DMAG significantly increased apoptosis cells at 75 nM (Fig. 5C).

### Changes in expression of RhoA and its downstream factors

**Figure 6:**
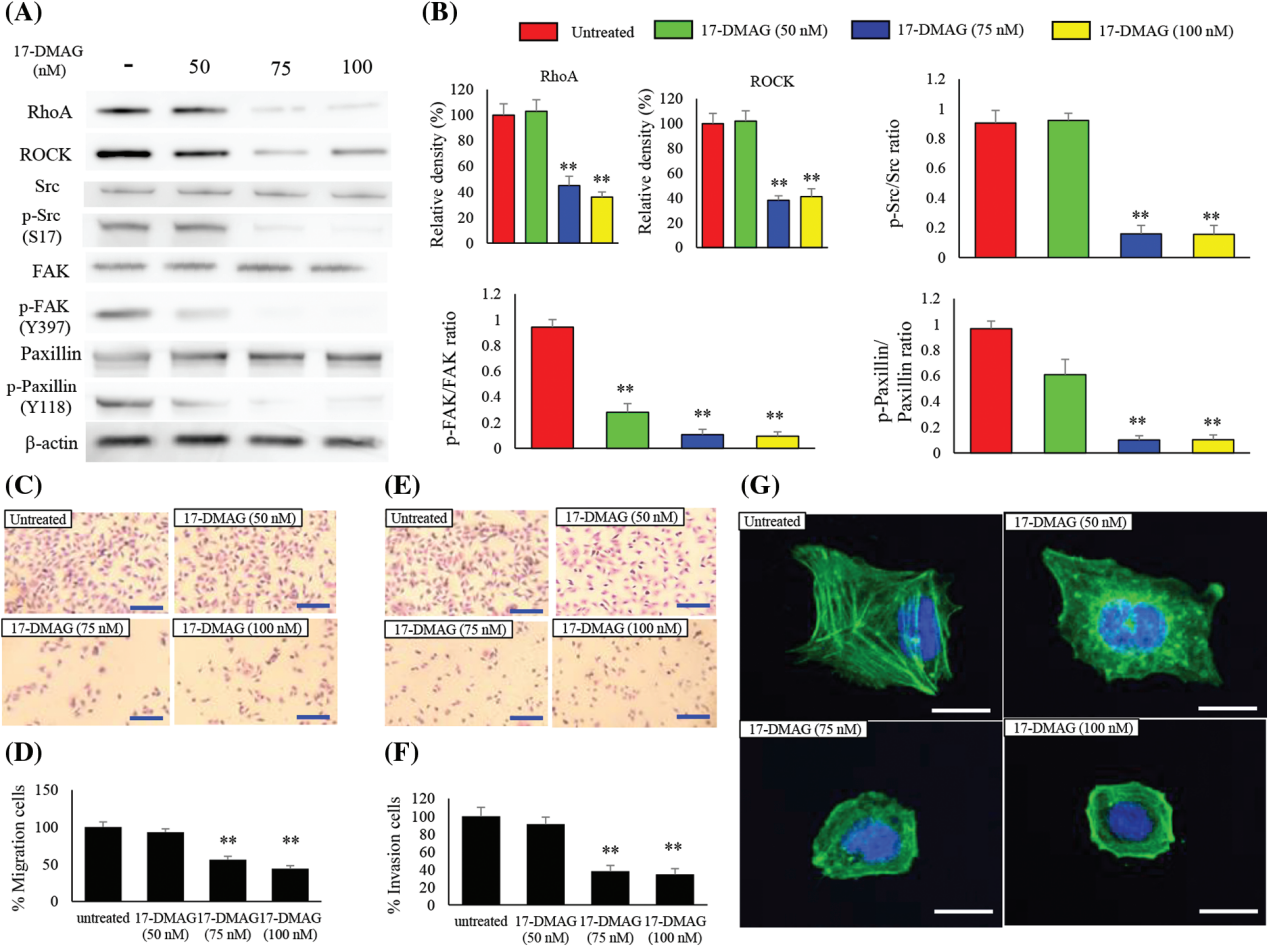
Effect of 17-DMAG on motility of MG63 cells. Changes in the expression of factors related to cell motility (A) and quantification of western blotting (B). Phosphorylation/total ratio was analyzed as quotient of phosphorylation *vs*. total protein expression. Migration of MG63 cells was assessed in each group at 24 h after 17-DMAG treatment. Scale bar: 40 μm (C). Quantification of the number of MG63 cells that crossed the membrane (D). A significant decrease in motility was found in the group administered 17-DMAG. (E) Invasion of MG63 cells was assessed in each group after 24 h. Scale bar: 40 μm. Quantification of the number of MG63 cells that crossed the membrane with Matrigel (F). Decreased migration ability was found in the group administered 17-DMAG. All data represent the mean ± SD from three independent experiments. **p < 0.01. The effect of 17-DMAG on actin fiber morphology was evaluated using immunofluorescent imaging (G). Confocal microscopy was used to confirm the distribution of F-actin and nucleus by Phalloidin and 4′6-diamino-2-fenilindol dihidrocloreto staining, respectively. Scale bars, 20 μm.

RhoA is a downstream signaling factor of MET and is intricately involved in cell motility [22,23]. We also investigated the effect of 17-DMAG on the expression of RhoA and the associated downstream factors, ROCK, Src, p-Src, FAK, p-FAK, paxillin, and p-paxillin (Fig. 6A). RhoA and ROCK expressions were significantly decreased in the treatment groups compared with the untreated group (*p* < 0.01). No significant difference was noted in Src, FAK, paxillin expressions among the untreated treatment groups. However, p-Src (S17), p-FAK (Y397), p-paxillin (Y118) expressions were significantly decreased in the treatment groups compared with the untreated group (*p* < 0.01) (Fig. 6B).

### 17-DMAG modulates the migration and invasion ability of osteosarcoma cells

To determine whether treatment of osteosarcoma cells with 17-DMAG influenced cell migration and invasion properties, we first performed a transwell motility assay with dose-dependent administration of 17-DMAG to MG63 cells (Fig. 6C). A statically reduced migration capacity was observed in the treatment groups compared with the untreated group (Fig. 6D). An invasion assay was also performed on the MG63 cells treated with 17-DMAG (Fig. 6E). The treatment groups showed a statically reduced migration capacity when compared with the untreated group. Hence, treatment with 17-DMAG altered the migration and invasion ability of osteosarcoma cells (Fig. 6F). To further evaluate the invasive ability of the osteosarcoma cells, we performed fluorescent staining of actin fibers. Notably, actin fibers were extended in the untreated cells, while cells treated with 17-DMAG exhibited a reduction in the extension of actin fibers in a dose-dependent manner (Fig. 6G).

### In vivo tumor bearing nude mice models

**Figure 7:**
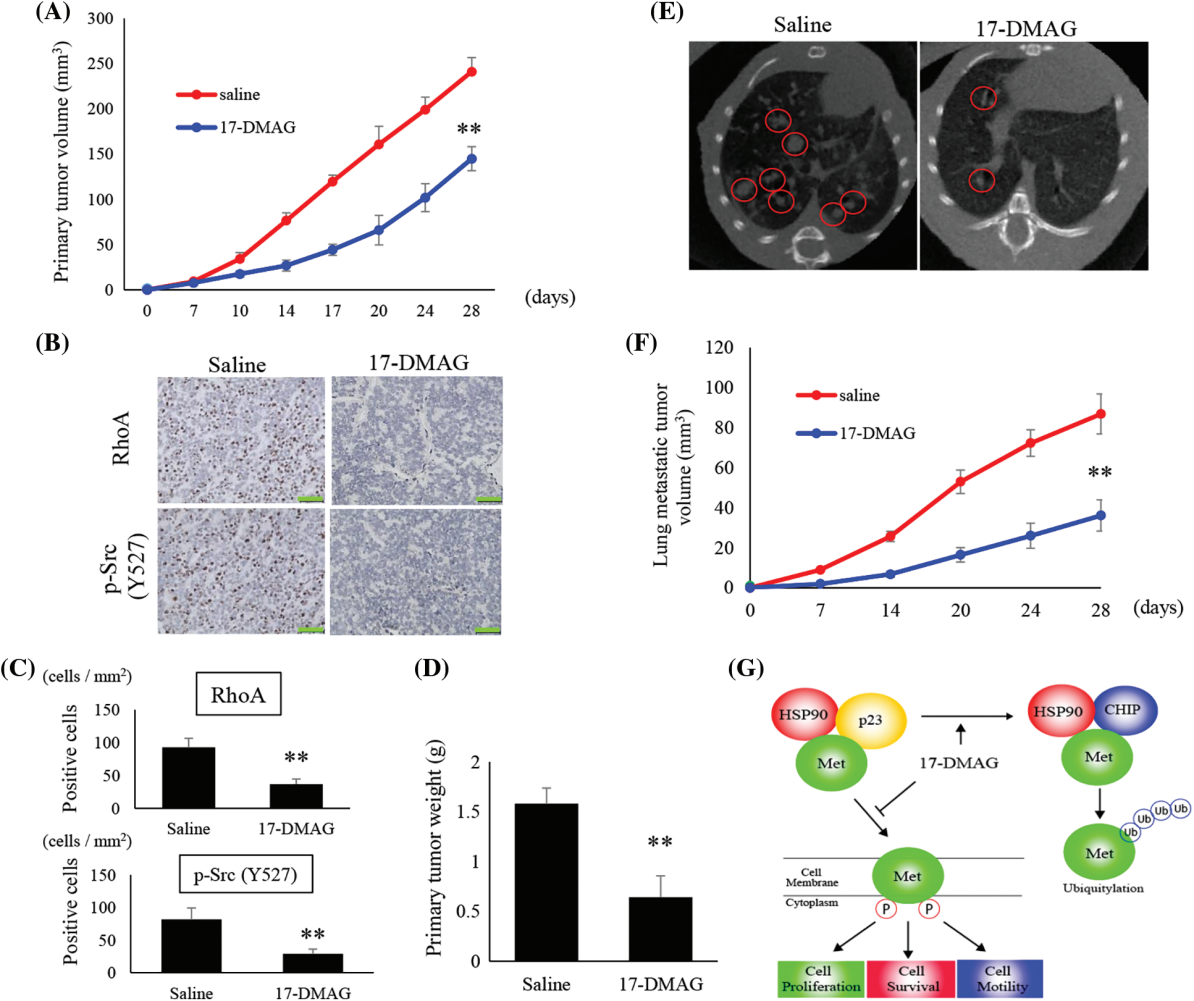
Suppression of osteosarcoma tumor growth by 17-DMAG. The tumor volumes in implanted mice were compared in each group (A). Immunohistochemistry image analysis for the expression RhoA and p-Src (Y527) in the saline and 17-DMAG-treated tumor sections. Scale bar: 50 μm (B). Quantification of the number of RhoA and p-Src positive cells per unit area (C). Treatment reduced the weight of the primary lesion (D). Lung metastases were evaluated by micro-CT. Lung metastatic lesions are circled in red (E). The volume was quantified to show the therapeutic effect of 17-DMAG (F). All data represent the mean ± SD from three independent experiments. *p < 0.05; **p < 0.01. Diagram summarizing the mechanism by which HSP90 inhibition suppresses the MET pathway and downstream signaling mediators (G). The figure demonstrates changes in the structural elements of the MET-HSP90 complex induced by administration of 17-DMAG.

We analyzed the tumors that developed in mice xenografted with MG63 cells from two treatment groups; saline-treated group, and mice treated with 17-DMAG. The size of tumors in mice inoculated with 17-DMAG (144.9 ± 13.2 mm^3^) was significantly smaller than that of tumors in mice from saline-treated groups (248.2 ± 14.9 mm^3^) (*p* < 0.01) (Fig. 7A). Immunohistochemical studies revealed that the expression of RhoA and p-Src (Y527) in the xenografted tumors was inhibited by 17-DMAG (Fig. 7B). The number of cells positive for RhoA expression was significantly reduced in mice treated with 17-DMAG (36.6 ± 8.1 cells/mm^2^) compared with that in the saline-treated groups (93.1 ± 13.8 cells/mm^2^) (*p* < 0.01). The number of cells positive for p-Src expression was also significantly reduced in mice treated with 17-DMAG (29.1 ± 6.6 cells/mm^2^) compared with that in the saline-treated group (81.2 ± 12.5 cells/mm^2^) (*p* < 0.01) (Fig. 7C). The weight of the resected tumor tissue was significantly smaller in the 17-DMAG group (0.64 ± 1.06 g) than that in the saline group (1.58 ± 0.21 g) (*p* < 0.01) (Fig. 7D). The volume of the lung metastases in each group was evaluated by micro-CT (Fig. 7E). Compared to that in the saline group (86.8 ± 10.1 mm^3^), a greater shrinkage of the lung metastases was observed in the 17-DMAG group (36.1 ± 7.8 mm^3^) (Fig. 7F). The overall results of this study show that MET protein complexes with HSP90 and p23, and then releases signals involved in cell proliferation, anti-apoptosis, and cell motility as a tyrosine kinase protein (Fig. 7G).

## Discussion

There have been several advances in the treatment of osteosarcoma over the past 20‒30 years. However, no effective treatment has been developed for patients resistant to the standard treatment procedures. Multiple studies have previously indicated the effectiveness of HSP90 inhibitors which could suppress the proliferation of cancer cells [24,25]. The antitumor effect of 17-DMAG has already been proven in other cancers, and a number of clinical trials are in progress. However, the role of 17-DMAG in the treatment of osteosarcoma remains to be elucidated [26,27].

HSP90 exhibits a wide range of functions as a molecular chaperone in normal cells [28]. This function is accelerated in cancer cells and is strongly involved in cell immortalization [29]. Thus, MET formed a complex with HSP90 in MG63 cells, and the administration of 17-DMAG resulted in dissociation of HSP90 and p23, coupled with binding to the ubiquitin ligase CHIP, and thus, ubiquitination of MET protein. Indeed, treatment of osteosarcoma cells with 17-DMAG resulted in reduced cell proliferation within a dose range of 10–100 nM. Hence, we ascertained that the optimal treatment concentration lies within this dose range. MET, a tyrosine kinase receptor, has previously been reported to have a clear correlation with the prognosis of hepatocellular carcinoma and stomach cancer [30,31]. MET is also an HSP90 client protein, and the function of HSP90 is intimately tied with the construction of the receptor structure [32,33]. We confirmed that administration of 17-DMAG in MG63 cells inhibited MET phosphorylation in a dose- and time-dependent manner. To determine the factors that contributed to reduced growth of osteosarcoma cells, we first assessed for retardation of the cell cycle. Notably, 12 h after administration of 17-DMAG to MG63 cells, the cell cycle progression was halted at the G2/M phase, indicating that treatment with 17-DMAG affected the early response in cell growth inhibition. In other cancers, inhibition of cell cycle progression was shown to be caused by the reduction of CCNB1 and CDK1 expression induced by the inhibition of HSP90 [34,35]. These results also correlated with prior reports demonstrating that inhibition of MET phosphorylation also inhibited PI3K and Akt phosphorylation, leading to the cessation of cell cycle progression [36,37]. Another factor that may contribute toward reduced cell proliferation is the induction of apoptosis [37]. We further showed that this effect was caused by reduced Akt phosphorylation resulting from reduced signaling from MET and weakened anti-apoptotic effects.

The MET signaling pathway is also involved in the motility of tumor cells and is particularly important for acquiring an invasive property via RhoA [38] and Src [39]. Indeed, administration of 17-DMAG to MG63 cells reduced the motility and invasiveness of the tumor and also suppressed the extension of actin filaments. Thus, it became clear that administration of 17-DMAG also reduced RhoA and Src signaling, indicating that both the proliferation and motility of tumor cells were significantly reduced. We successfully demonstrated a tumor regression effect in the group of mice administered 17-DMAG. Furthermore, MET and Akt phosphorylation was reduced only in the tissue resected from the tumors obtained from the 17-DMAG treatment group.

The limitation of this experiment is that we have not analyzed the three-dimensional structure of proteins. We have premised that the inhibition of HSP90 by 17-DMAG impairs the correct protein folding function. However, we have not directly analyzed whether the MET protein of MG63 cells has an inappropriate structure. Adverse events are an important challenge in the search for GA treatments. Adverse events associated with 17-DMAG include fatigue, anorexia, nausea, blurred vision, and musculoskeletal pain [27,40]. A clinical trial of an HSP90 inhibitor found that the dose of the HSP90 inhibitor could be reduced by combining with trastuzumab [26], as this makes it possible to suppress undesirable adverse events, considering that combination therapy may also be effective for osteosarcoma cells. Studies investigating the differences in the effects of 17-DMAG on various isoforms of HSP90, such as HSP90 aa1, ab1, and TRAP1 have not been conducted. In our study, we specifically investigated the effects of 17-DMAG on HSP90 aa1, as its mechanism of action involves inhibiting the ATP-binding site of the N-terminal domain (NTD) of HSP90 [41]. It is therefore presumed that 17-DMAG shares a common mechanism of action across HSP90 isoforms. Thus, it is likely that the potency of 17-DMAG is similar among different isoforms of HSP90. Recognition of extracellular HSP90 (eHSP90) expressed on the surface of cancer cells by dendritic cells, leading to the activation of anti-tumor immunity has been reported [42]. Considering that the presence of eHSP90 on the cell membrane through eHSP90 ab1 can activate anti-tumor immunity, a disadvantageous effect that the inhibition of eHSP90 by 17-DMAG may have on tumor immunity cannot be disregarded.

On the other hand, the role that eHSP90 plays in regulating tumor cell motility and enhancing metastatic potential has been reported, including increased cell adhesion and motility through integrin-mediated signaling [43], enhanced cell invasion through binding with HER2 and ErB3 [44], and increased cell invasion through actin rearrangement [45]. The inhibition of HSP90 by 17-DMAG is considered more useful as an anti-cancer agent, as it does not only inhibit cell proliferation but also suppresses metastatic potential.

In summary, we confirmed that treatment with 17-DMAG results in a CHIP-MET protein complex, resulting in the modification and destruction of ubiquitin and reducing the signal for cancer progression. Previous research has also reported that PI3K and Akt signaling is controlled by the MET signaling pathway [46,47]. While there have been previous reports of the anti-cancer effects of 17-DMAG [48,49], this is the first study to demonstrate the ubiquitination of MET protein induced by 17-DMAG in osteosarcoma cells. As MET is intimately involved in resistance to chemotherapy, we predict that 17-DMAG may prove to be a promising therapeutic agent for treating osteosarcoma.

## Data Availability

All data analyzed during this study are included in this manuscript. The cDNA array data was deposited in Gene expression Omnibus (GEO). The GEO reference number is GSE70415 and URL to access our raw data is below: http://www.ncbi.nlm.nih.gov/geo/query/acc.cgi?acc=GSE70415.
